# Assessing Mental Health for China’s Police: Psychometric Features of the Self-Rating Depression Scale and Symptom Checklist 90-Revised

**DOI:** 10.3390/ijerph17082737

**Published:** 2020-04-16

**Authors:** I-Hua Chen, Chung-Ying Lin, Xia Zheng, Mark D. Griffiths

**Affiliations:** 1 College of Education Science, Minnan Normal University, Zhangzhou 363000, China; 2 Department of Rehabilitation Sciences, Faculty of Health and Social Sciences, The Hong Kong Polytechnic University, Hung Hom, Hong Kong; cylin36933@gmail.com; 3 Mental-Health Education Center, Nanchang University, Nanchang 330000, China; zhengxia@ncu.edu.cn; 4 International Gaming Research Unit, Psychology Department, Nottingham Trent University, Nottingham NG1 4FQ, UK; mark.griffiths@ntu.ac.uk

**Keywords:** Zung Self-Rating Depression Scale (Zung SDS), Symptom Checklist (SCL-90-R), psychometrics, police, police mental health

## Abstract

Police mental health is important because police officers usually encounter stressors that cause high levels of stress. In order to better understand mental health for Chinese police, the Zung Self-Rating Depression Scale (SDS) and Symptom Checklist 90-Revised (SCL-90-R) are commonly used in mainland China. Unfortunately, both the SDS and SCL-90-R lack detailed information on their psychometric properties. More specifically, factor structures of the SDS and SCL-90-R have yet to be confirmed among the police population in mainland China. Therefore, the present study compared several factor structures of the SDS and SCL-90-R proposed by prior research and to determine an appropriate structure for the police population. Utilizing cluster sampling, 1151 traffic police officers (1047 males; mean age = 36.6 years [SD = 6.10]) from 49 traffic police units in Jiangxi Province (China) participated in this study. Confirmatory factor analysis (CFA) with Akaike information criterion (AIC) was used to decide the best fit structure. In the SDS, the three-factor model (first posited by Kitamura et al.) had the smallest AIC and outperformed other models. In the SCL-90-R, the eight-factor model had the smallest AIC and outperformed the one-factor and nine-factor models. CFA fit indices also showed that both the three-factor model in the SDS and the eight-factor model in the SCL-90-R had satisfactory fit. The present study’s results support the use of both SDS and SCL-90-R for police officers in mainland China.

## 1. Introduction

As a force for maintaining social stability, the police are the executors of maintaining public order and protecting the safety of citizens. In order to implement relevant laws and regulations in society, the police often work overtime and face unexpected stressful conflicts. Consequently, the police may experience negative emotions that contribute and/or cause mental health problems [1,2]. According to a report from the Occupational Disease Intelligence Network for Surveillance of Occupational Stress and Mental Illness, the proportion of police officers suffering from psychological disorders ranks in the top three among various occupations [3]. This situation is severe in China’s rapidly developing society because the police have to undertake increasingly unfamiliar tasks. According to a survey of 14,664 young police officers in China, 38.9% were reported as having likely mental health problems, which is significantly worse than that of ordinary adults [4].

In order to assess mental health among the police, the Zung Self-Rating Depression Scale (SDS) and Symptom Checklist 90-Revised (SCL-90-R) are commonly used [5,6,7,8,9,10,11]. However, previous studies rarely report the validity of the SDS or SCL-90-R among police. Previous studies have examined linguistic validity (e.g., [7,9]) and internal reliability (e.g., [11]), but such information alone is insufficient for healthcare providers or researchers to argue that the instruments are psychometrically robust and valid. Given that these two scales are of the most common measures utilized in studies of police officer’s mental health problems, more caution is warranted concerning the psychometric quality of these two tools, and stronger empirical evidence is needed.

In addition to the lack of research examining the psychometric properties of these instruments in this target population (i.e., police), the SDS and SCL-90-R are still controversial. The SDS, developed by Zung in 1965 [12], is a valid and sensitive measure in assessing clinical severity for patients with depression [13]. However, there have been some concerns in using SDS because the specification of factor structure for SDS is yet to be determined. The original SDS comprised 20 items with three dimensions [12], which are pervasive affect (e.g., crying spells), physiological equivalents (e.g., insomnia), and psychological equivalents (e.g., hopelessness). However, inconsistent factor structures have been found. Passik et al. reported a four-factor model in a population of cancer patients [14], Kitamura et al. [15] reported a three-factor model among 28,588 first-year university students, and Shafer [16] reported another three-factor model, of which items were embedded differently than the three-factor models proposed by Zung [12] and Kitamru et al. [15]. In Shafer’s study [16], the effect of item wording resulted in three factors comprising positive, negative, and somatic symptoms.

The SCL-90-R is a 90-item self-reported inventory developed by Derogatis et al. [17]. The SCL-90-R uses a nine-factor model to assess psychological symptoms and psychological distress. The nine factors proposed by Derogatis et al. [17] are somatization (SOM), obsessive-compulsive (O-C), interpersonal sensitivity (I-S), depression (DEP), anxiety (ANX), hostility (HOS), phobic anxiety (PHOB), paranoid ideation (PAR), and psychoticism (PSY). Although SCL-90-R has been widely used among various groups, problems with the SCL-90-R have been identified. More specifically, the subscales do not adequately distinguish clinical diagnoses [18], subscales are rarely discriminated apart from one another [19], and the scale as a whole has limited validity as a clinical measure [20]. Inconsistent factor structures have also been found in the SCL-90-R. For example, Arrindell and Ettema [21] proposed an eight-factor structure for the SCL-90-R (i.e., agoraphobia [AGO], anxiety [ANX], depression [DEP], somatic complaints [SOM], cognitive-performance deficits [COG], interpersonal sensitivity and mistrust [I-S], acting-out hostility [HOS]). Xie and Dai reviewed the SCL-90-R studies and asserted that the unstable factor structure was the main controversy [22]. They suggested that examination of the reliability and validity of SCL-90-R among different populations is necessary [22].

Being in good mental health is a prerequisite for ensuring that police officers can perform their role in maintaining social stability. Therefore, it is important to have a valid measurement tool to more accurately assess their mental health. If SDS and SCL-90-R are utilized to investigate police mental health, evidence concerning the two scales’ psychometric characteristics and robustness should be thoroughly understood. The purpose of the present study was to carry out a detailed examination of the psychometric characteristics of SDS and SCL-90-R among Chinese police officers. More specifically, the controversy concerning the factorial validity for SDS and SCL-90-R was addressed. The SDS has items embedded in the somatic factor that are psychometrically unstable across different populations [15], and the factor structure might change simply as an artifact of wording changes [16]. The SCL-90-R has subscales which are highly correlated, making it difficult to discriminate between the factors.

In addition to the factorial validity, the present study also tested whether the responses to the two scales among police officers can be related to their coping styles when facing pressure. In social life, all individuals inevitably face pressure, which usually impacts negatively on their mental health, and arguably more so among police officers. However, the same level of stress has different influences on individuals because of various coping styles [11,23]. Therefore, if the relationship between the coping style of the police when facing pressure and their mental health problems can be established, it will provide better evidence for intervention and prevention policies in everyday practice. The findings of the present study are expected to provide evidence of the reliability and validity of these two tools, and the findings are expected be helpful for subsequent research concerning police mental health issues if the two tools are validated.

## 2. Materials and Methods

### 2.1. Participants and Procedure

In the present study, cluster sampling was used to distribute questionnaires, including a background information sheet, SDS, SCL-90-R, and the Coping Style Questionnaire (CSQ), to 1218 traffic police officers from 53 traffic police units in Jiangxi Province (China). After removing invalid responses, 1151 participants (males = 1047; 91.0%) from 49 units remained for further analyses. The average age of the participants was 36.6 years (SD = 6.10) and had worked in the police force for an average of 12.24 years (SD = 4.45). Most of the participants were married (*n* = 948; 82.4%), followed by unmarried (*n* = 166; 14.4%) and divorced (*n* = 37; 3.2%). Most of the participants had a Bachelor’s degree (*n* = 1092; 95.0%) and the rest had a Master’s or a Doctoral degree. A total of 462 participants (40.1%) had leadership status within their unit.

This study was approved by the research ethics committee of the local university in Jiangxi Province (IRB No. 2019xx0310TP). With the assistance of the authority of the Jiangxi Provincial Traffic Police, a qualified counselor and the research team distributed the questionnaires to the traffic police units. Before participating in the survey, the research team ensured to the participants that their data would be under high privacy protection, and that their personal information and results would not be given to their line managers. After investigation, the counselor gave feedback to the participants individually concerning the results. For those with likely mental health problems, they were referred to the psychological assistance department for consultation and interviews before returning to the Public Security Department of Jiangxi Province.

### 2.2. Measures

The SDS [12] comprises 20 items that evaluate the symptoms of depression. Participants rate each item according to how they felt during the preceding week. Item responses are rated on a four-point rating scale (1–4) with higher scores corresponding to more frequent symptoms. Higher SDS scores indicate higher levels of depression. The sum of the scores of the 20 items is the total score, and the total score is multiplied by 1.25 to provide the SDS index score. According to the Chinese SDS manual [24], those scoring (i) 50–59 are classed as having mild depression; (ii) 60–69 are classed as having moderate-to-marked depression; and (iii) those scoring 70 and over are classed as having severe-to-extreme depression. Cronbach’s α was 0.83 and McDonald’s ω was 0.84 for the total SDS score in the present study.

The SCL-90-R assesses the severity of psychological distress in the past week using a five-point Likert-scale (from “not at all” to “severe”). According to the norm of Chinese SCL-90-R version [22], the scores of the 90 items are summed up to obtain the total score. The factor scores are obtained by summing the item scores embedded within the same factor. Total scores more than 160, 200, and 250 indicate presence of psychological distress, moderate distress, and severe distress, respectively. When a factor score is ≥2, it indicates that the participants have more serious symptoms in that factor. Similar to the SDS, there has been no information on factorial validity of SCL-90-R among Chinese police. Guo et al. reported that the overall Cronbach’s α of SCL-90-R was 0.89 in the study of prison police [11]. Cronbach’s α was 0.98 and McDonald’s ω was 0.89 for the total SCL-90-R score in the present study.

The CSQ consists of 62 items comprising six subscales. The scale assesses individual coping styles when facing pressure. Each subscale represents a specific coping style. The content of item describes a specific way to cope with pressure (e.g., focus on solving problems, just giving up, asking others for help to overcome difficulties) and the scoring of CSQ is dichotomous (either agreeing [1 point] or disagreeing [0 points] with the statement). According to the instruction manual of CSQ [25], the scores of each subscale are added up and then divided by the number of items to obtain the factor score. A higher factor score indicates that individuals are more inclined to use a specific coping style. In the present study, the Cronbach’s α of problem-solving, self-blame, help-seeking, illusion, avoidance, and rationalization were 0.75, 0.81, 0.64, 0.70, 0.69, and 0.62, respectively, and the McDonald’s ω for the aforementioned factors were 0.77, 0.82, 0.67, 0.69, 0.68, and 0.64, respectively.

### 2.3. Data Analysis Strategy

Using LISREL 8.80, confirmatory factor analysis (CFA) was applied to estimate the model fit of data to the proposed factor structures. Using police officers’ scores on the SDS and SCL-90-R, the study aimed to identify the most suitable factor structure among several competing models. For the SDS, a number of models were proposed. The one-factor model was chosen as the baseline model followed by Schotte et al.’s two-factor model [26], Zung’s three-factor model [12], Kitamura et al.’s three-factor model [15], Shafer’s three-factor model [16], and Passik et al.’s four-factor model [14]. Among these, the models of Schotte et al. and Shafer both reflect item wording artifacts, and these structures are not actually substantively symptom domains of depression. This is because half the items in the SDS have positive wording (e.g., Item 2 “feel good in the morning” is worded in a positive way and then the score of this item is reversed indicating “feel worse in the morning”), and half of the items have negative wording. This special characteristic might result in the participant being affected by artificial factors of positive and negative wording (i.e., wording effect). If these two models fit well with the data, it is concluded that an item-wording effect exists for the SDS. In addition, it is worth mentioning that although the previous literature did not indicate that there was a potent general factor in the SDS, in order to be consistent with the analysis of SCL-90-R below, a bi-factor model was also tested for the SDS in model fitting.

Similarly, for SCL-90-R, a number of models were proposed. A one-factor model was chosen as the baseline model followed by Derogatis’s nine-factor model [17], Arrindell and Ettema’s eight-factor model [21], and the second-order factor model with overall general distress factor corresponding to the eight-factor and nine-factor models. In addition, the bi-factor model for the eight-factor and nine-factor models was proposed because recent literature indicates that using the bi-factor model for SCL-90-R can help to determine whether each subscale has sufficient unique factorial validity, and that this information cannot be obtained by simply using the higher-order factor model [27,28,29]. Among the first five models (i.e., one-factor, nine-factor, eight-factor, and the two second-order models), it was investigated whether the eight-factor model was better than the original nine-factor model and whether it was more suitable for Chinese police officers. It was also investigated whether a hierarchical factor model should be used for the SCL-90-R. Utilizing the bi-factor model, it can be judged whether SCL-90-R has a significant general factor for police officers, and after considering the general factor, whether each dimension has the theoretical influence as expected.

CFA for fit estimation of indices were the chi-square, the comparative fit index (CFI), the non-normed fit index (NNFI), the root-mean-square error of approximation (RMSEA), and the standardized root-mean-square residual (SRMR). RMSEA values of 0.08 or lower, SRMR values of 0.09 or lower, CFI values of 0.90 or higher, and NNFI values of 0.90 or higher are considered acceptable [30]. Akaike information criterion (AIC) was used to compare the models with acceptable fit indices and decide the best model fit. More specifically, smaller AIC indicates a better fit [31]. The composition reliability (CR) and average variance extracted (AVE) were also calculated. According to Fornell and Larcker [32], convergent validity is supported if the CR is higher than 0.5 and AVE is higher than 0.7 for each construct. Moreover, AVE should be larger than *r*^2^ (squared correlation between the two factors) to support discriminant validity.

In order to quantify the potent degree of the general factor in the bi-factor model, the indices of explained common variance (ECV), percentage of uncontaminated correlations (PUC), and Omega Hierarchical (OH) were used. ECV refers to the ratio of the variance explained by the general factor to the whole explained variance by model. PUC refers the ratio of the number of the uncontaminated correlations to the number of the unique correlations. OH for the general factor refers to the percentage of the variance in the total scores that can attributed to the general factor, and the specific factor refers to the proportion of reliable variance of a subscale after partitioning out the variance attributed to the general factor. If the general factor of OH is >0.80, a significant general factor can be considered as existing. If both ECV and PUC are >0.70, the relative bias will be slight, and the common variance can essentially be attributed to the general factor [33].

**Figure 1:**
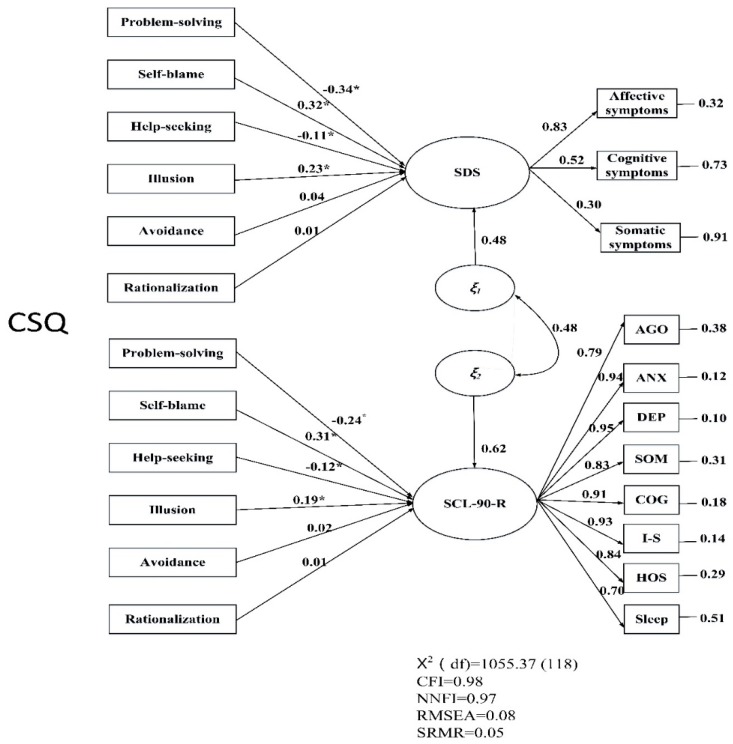
SEM of Coping Style Questionnaire (CSQ), Self-Rating Depression Scale (SDS), and Symptom Checklist 90-Revised (SCL-90-R). The six coping styles of CSQ were as exogenous variables by summing the scores of the corresponding items. The factor structure of SDS used Kitamura et al.’s [15] three-factor model and SCL-90-R used Arrindell and Ettema’s [21] eight-factor model. Note. * *p* < 0.05.

To examine the criterion validity, the six coping styles of CSQ were used as exogenous variables to test their influence on the SDS and SCL-90-R with structural equation modeling (SEM). The best-fit factor structures of the SDS and SCL-90-R were used in the SEM to represent their measurement parts (Figure 1). The subscale scores on the SDS, SCL-90-R, and CSQ were treated as the indicators. The residuals of SDS and SCL-90-R were set to be correlated because of the high overlap. The overall model fitting with the aforementioned indices, CFI, NNFI, RMSEA, and SRMR, was examined first. The CR and AVE were then used to judge the convergent validity of the SDS and SCL-90-R in the same model. It was anticipated that similar results would be obtained to those shown in each individual CFA. After ensuring the overall model fitting and the quality in the measurement part, path coefficients between CSQ with SDS and SCL-90-R were examined to understand the criterion validity. More specifically, when police officers face pressuring events that they are accustomed to responding to actively (e.g., problem solving and seeking assistance), their psychological distress will be low. Conversely, adopting emotional-focused coping styles (e.g., self-blame, illusion, avoidance, and rationalization) may lead to police officers accumulating negative emotions and subsequently lead to high levels of psychological distress.

## 3. Results

### 3.1. Descriptive Statistics and Correlation Analysis

Descriptive statistics of SDS, SCL-90-R, and CSQ scores are presented in Table 1. The percentages of participants with depression or psychological distress were 59.6% using SDS index (i.e., index higher than 50) and 52.0% using the total score of SCL-90-R (i.e., score higher than 160). The percentages of a severe degree of mental health problems were 4.3% using SDS index (i.e., higher than 70) and 11.1% using SCL-90-R (i.e., higher than 260). The relatively higher dimensions of the subscales in the SCL-90-R were O-C and DEP (i.e., both factor scores were higher than 2).

The result of Pearson correlations (Table 1) showed that there was a significant positive correlation between SDS and SCL-90-R (*r* = 0.66; *p* < 0.01). Moreover, the SDS was significantly positively correlated with all SCL-90-R subscales (*r* scores ranging from 0.50 to 0.66). Among these subscales, the highest *r* coefficient was SDS and DEP in the SCL-90-R. The six types of coping styles were all significantly correlated with SDS and SCL-90-R scores. More specifically, problem-solving and help-seeking were negatively correlated with SDS (problem-solving: *r* = −0.42, *p* < 0.01; help-seeking: *r* = −0.28, *p* < 0.01) and SCL-90-R (problem-solving: *r* = −0.36, *p* < 0.01; help-seeking: *r* = −0.26, *p* < 0.01). Self-blame, illusion, avoidance, and rationalization were positively correlated with SDS and SCL-90-R (*r* scores ranging from 0.31 to 0.52; *p* < 0.01).

### 3.2. Confirmatory Factor Analysis

In the CFA of the SDS, the one-factor model and Zung’s original three-factor model had unacceptable fit and did not meet the standards in the all indices. The other four models had acceptable model fits (see Table 2; the coefficients of factor loading, measurement error, and relationship between latent variables are marked in the figure of each model in the Supplementary Material). CFI and NNFI were all higher than 0.90, RMSEA ranged from 0.054 to 0.067, and SRMR ranged from 0.064 to 0.081. AIC further suggested that Kitamura et al.’s three-factor model was the best fitting model. The bi-factor model with Kitamura et al.’s three-factor model was then tested to see whether a potent general factor existed in the SDS. The results showed that the ECV and OH were 0.52 and 0.65, both below the recommended cut-off points. Therefore, there was no significant general factor in the scores of the SDS. Among the three factors, compared to cognitive and somatic symptoms, the ECV and OH were relatively low (i.e., ECV for cognitive and somatic symptoms were 0.19 and 0.06; OH for cognitive and somatic symptoms were 0.41 and 0.27), the ECV and OH of affective symptoms were 0.74 and 0.65, indicating a unique contribution of explained variance after considering the effect of the general factor.

Because the best model was Kitamura et al.’s three-factor model, Table 3 demonstrates the standardized factor loading based on this factor structure. This information was subsequently used to calculate CR and AVE. The results show that there was only one factor loading less than 0.5 in each factor. The CR of affective, cognitive, and somatic symptoms were 0.86, 0.80, and, 0.72. The AVE for these three symptoms were 0.44, 0.42, and 0.38. Therefore, the convergent validity was not fully supported because all AVEs were less than 0.50. The reasons for unsatisfactory AVE results might be the low factor loading from Items 8, 2, and 7 (all <0.4; Table 3). Regarding discriminant validity, the correlation between affect with cognitive symptoms was 0.49, the correlation between affect with somatic symptoms was 0.34, and the correlation between cognitive with somatic symptoms was 0.78. The AVE of affective symptoms was higher than the *r*^2^ of this variable and the other two constructs (i.e., 0.24 and 0.12), but the AVE of cognitive and somatic symptoms was lower than the *r*^2^ of these two constructs (i.e., 0.61). Therefore, discriminant validity was not supported in the SDS.

Table 3 also shows that after partitioning out the influence of general factors, the specific factor loadings among affective symptoms were not substantially changed. However, the factor loadings in the other two symptoms were substantially changed, especially the cognitive symptoms having five factor loadings out of six below 0.20 (even producing unreasonable negative values) when considering the general factor. Therefore, although a significant general factor did not exist across three factors in the SDS, there was still a moderate common factor between cognitive and somatic symptoms, which caused substantial changes in the loadings of the specific factor. The result echoes the poor discriminant validity among these two factors (mentioned in the previous paragraph).

In the CFA of SCL-90-R, apart from the two second-order models not converging with the data, the other five models tested all showed acceptable fit, although RMSEA was slightly high in the one-factor model (i.e., RMSEA = 0.093; Table 2). Among the one-factor, nine-factor, and eight-factor models, the eight-factor model had the smallest AIC. In the bi-factor model for nine-factor and eight-factor models, the ECV and OH of the general factor in the nine-factor model were 0.87 and 0.98. The ECV and OH in the eight-factor model were 0.82 and 0.97. Given that PUC was at high levels for both (i.e., for nine-factor model PUC was 0.89, and it was 0.86 for eight-factor model), it can therefore be concluded that the total scores of SCL-90-R were mainly influenced by a potent general factor irrespective of whether it was an eight-factor or nine-factor structure.

Table 4 provides the standardized factor loading of SCL-90-R with the best fitting model (i.e., eight-factor model and this model in a bi-factor model). The results showed that the factor loadings were all above 0.50. CR and AVE of these eight factors were further calculated. The results showed that CRs were higher than 0.80 (ranging from 0.83 to 0.95) and AVEs were higher than 0.50 in all domains (ranging from 0.50 to 0.62), except for COG (AVE = 0.49), indicating satisfactory convergent validity. However, the discriminant validity did not meet the expected standard because of the high correlations between the factors (i.e., 20 of the 27 correlation coefficients were more than 0.8).

As for the factor loading of the subscales in the bi-factor model, after considering the general factor, the loading from each symptom was lower than that of the original eight-factor model while the loadings on the general factor were strong (i.e., all >0.50). It is noted that some loadings were close to zero and there was coexistence of positive and negative correlations in the ANX, DEP, COG, and I-S subscales. These results show that, after considering the general factor, the subscales of ANX, DEP, COG, and I-S were seriously ill-defined. The OH for these four specific factors were less than 0.10 (especially for ANX and DEP, both less than 0.03), indicating that the contribution of these specific factors is negligible.

### 3.3. Structural Equation Modeling

Finally, Figure 1 shows the criterion validity of the SDS and SCL-90-R. Given that Kitamura et al.’s three-factor model and Arrindell et al.’s eight-factor model had the best fit for the SDS and SCL-90-R, respectively, these two factor structures were used in the measurement part of the SEM. In the model, averaged scores of each factor were used as indicators of the latent variable, SDS, and SCL-90-R. The results showed that the overall model had acceptable fit (CFI = 0.98, NNFI = 0.97, RMSEA = 0.08, and SRMR = 0.05). In addition to the overall model fit, the quality of the measurement part was supported. For the SCL-90-R, the convergent validity was satisfactory in that CR and AVE were 0.96 and 0.75. However, CR and AVE in the SDS were 0.58 and 0.35, indicating poor convergent validity. In general, whether the SDS and SCL-90-R were tested in the same model or in the separate CFA, the judgment concerning convergent validity was similar.

The SEM results further showed that the relationship between six coping styles and the two scales (SDS and SCL-90-R) shared the same patterns. Problem-solving and help-seeking were negatively and significantly associated with the SDS (problem-solving: *r* = −0.34, *p* < 0.01; help-seeking: *r* = −0.11, *p* < 0.01) and SCL-90-R (problem-solving: *r* = −0.24, *p* < 0.01; help-seeking: *r* = −0.12, *p* < 0.01), while the two scales were positively and significantly associated with self-blame (SDS: *r* = 0.32, *p* < 0.01; SCL-90-R: *r* = 0.31, *p* < 0.01) and illusion (SDS: *r* = 0.23, *p* < 0.01; SCL-90-R: *r* = 0.19, *p* < 0.01). Therefore, the criterion validity hypothesis proposed in the present study was supported, except that avoidance and rationalization coping styles were not significantly associated with SDS and SCL-90-R .

## 4. Discussion

### 4.1. General Discussion

Both the SDS and SCL-90-R have a long history in the assessment of mental health problems. However, their psychometric features among police officers has been unclear. Therefore, in order to address this literature gap, Chinese police were sampled to thoroughly analyze the psychometric characteristics of these two scales. In addition, given that these two measures still had some controversies in terms of factorial validity, the factor structures were also rigorously tested. The results showed that the reliability of both the SDS and SCL-90-R was good. Moreover, the latent variables of the two scales were significantly related to the coping style adopted by police officers when they were under pressure, which provided reasonable evidence of criterion validity.

The controversy concerning the SDS lies in the factor structure, including methodological issues relating to item wording [16], and the symptoms of depression may change due to different characteristics of populations, leading to unstable somatic symptoms [15]. The findings indicated that the wording effect also existed in the Chinese SDS, at least among the population of police officers sampled. Moreover, most of the somatic symptoms of depression can be manifested in this study, except for the low factor loading of Item 7 (“losing weight”). This finding is in line with Pérez et al. [34], that the two-factor model (i.e., positive and negative wording) and the substantive symptom model (i.e., affect, cognitive, and somatic symptoms) can be both supported among healthy individuals but not in unhealthy individuals. More specifically, in this study, Schotte et al.’s [26] two-factor model and the Shafer’s [16] three-factor model both fitted the data well, and these models reflected the artifactitious factors of positively and negatively worded items. As for the best fitting model, Kitamura et al.’s [15] three-factor model had the lowest AIC. It also indicated three clear symptoms of depression and had acceptable factor loading (except for a few items). Therefore, this model is recommended for future use in studies if the targeted population is police officers. According to the results of the bi-factor model used in the present study, there was no obvious general factor in Kitamura et al.’s three-factor model. Among the three factors, affective symptoms had sufficient contribution to the explained variance. Therefore, this subscale may be used alone in the clinical diagnoses.

Although further progress has been made in the verification of SCL-90-R’s factor validity, these results have not been applied to the Chinese version of SCL-90-R. To the best of the authors’ knowledge, the number of studies using the SCL-90-R to assess police officers’ mental health is much higher than that those using the SDS in China. Unfortunately, there are no reports of the psychometric properties of the SCL-90-R in the 40+ studies on Chinese police that the authors have reviewed prior to this study. A serious concern is that most of these studies analyzed the subscale scores of nine dimensions (e.g., [9,35]). However, it should also be noted that these studies were carried out before the nine-factor model had been confirmed. In fact, comparing to the original nine-factor model, the present study found that eight-factor model appeared to be more suitable for Chinese police officers. Consequently, the Chinese version manual of SCL-90-R should be revised by adding a description concerning the eight-factor model structure. Moreover, the present study showed that irrespective of whether the model was nine-factor or eight-factor, their second-order model could not be converged with the data. Given that this result is different from some previous studies [27,28,29], future studies are needed to investigate whether the second-order model does or does not fit for police samples.

Regarding the application of bi-factor model, the present study found that after considering the influence of a general factor, some subscales contributed very little to the overall explained variance in the model, especially for the depression and anxiety symptoms. This result shows that if the scores on these two subscales were calculated separately and were used as the basis of clinical diagnosis, such diagnoses may be incorrect. In the case of empirical research, van der Velden et al. [7] assessed police mental health problems utilizing the subscales of depression, anxiety, and hostility of SCL-90-R. In their study, if the scores in any subscales exceeded a cut-off value, a police officer would be classified as having mental health problems. However, such practice may be inadequate based on the results reported here because there is large doubt as to whether these two dimensions really reflected the two theoretical constructs that were supposedly examined.

Finally, as for the evidence of criterion validity in this study, the higher the coping style scores of problem-solving and seeking assistance when the participants faced stressful incidents, the lower the degree of depression (SDS score) and the psychological distress (SCL-90-R score). The higher the score on self-blaming and illusion was, the higher the scores on the SDS and SCL-90-R. This result supports the proposed hypothesis. According to the definition of these coping styles, the former (i.e., problem-solving and help-seeking) are problem-oriented coping styles, and the latter is an emotion-oriented coping style that was discussed within Lazarus and Folkman’s stress-response theory [36]. Some studies have shown that problem-oriented coping styles can positively predict adaptive outcomes (e.g., life quality [37]), and emotion-oriented coping styles can impact negatively on mental health (e.g., depression [38]). The results of this study are consistent with these studies.

### 4.2. Practical Implications

The findings of the present study have two major practical implications. The first is the robustness of the factor structure tested, which can be used as a reference for scholars who intend to use SDS and SCL-90-R in future investigations of police officers’ mental health. This allows them to confidently assess such variables given the reliability and validity of these two scales. Secondly, the scores obtained on the SDS and SCL-90-R showed that over half of the participants had depression and psychological distress. This result is consistent with a meta-analysis reviewing empirical studies using SCL-90-R from 1996 to 2015 among Chinese police officers [39]. However, traffic police in the present study had relatively higher percentages (59.6% from SDS and 52.0% from SCL-90-R) of depression and psychological distress compared to police officers working in counter-terrorism [9] and prisons [11] with the same measures. The mental health issues of traffic police are often not paid attention to because they seldom face violence directly and usually face fewer emergencies than other types of police officers. However, as the importance of traffic laws increases and the number of vehicles on roads grows larger, traffic police may experience heavier working loads. Some Chinese studies have shown that Chinese traffic police are often abused by citizens because of traffic disputes (and may even be physically assaulted) [40,41]. Moreover, traffic police are less likely to be promoted as compared to other types of police officers [40,41]. As citizen complaints (from the issuing of ticket fines) and traffic disputes increase, and considering their lower promotion opportunity than other types of police officers, traffic police may suffer reduced self-esteem or job-esteem and have subsequent impacts on psychological health. According to the findings here, mental health concerns among traffic police should not be ignored either now or in the future.

### 4.3. Limitations and Future Research Directions

There are some limitations in the present study that should be taken into account when interpreting the findings. First, the participants in this study were limited to traffic police, and we did not explore whether different police officer types responded differently when using the SDS and SCL-90-R. With reference to a previous study by Li et al. [39], different types of police (such as riot police, general civilian police, prison police, and the traffic police) had significantly different overall psychological distress levels when assessed using the SCL-90-R. Therefore, testing the measurement invariance of SDS and SCL-90-R among different types of police officers is recommended for future research. Second, the SDS had several items with very low factor loadings (i.e., Item 8 “Constipation”, Item 2 “Worse in the morning”, and Item 7 “Weight loss”), which also led to problematic convergent validity and unsatisfactory discriminant validity. Future research could consider omitting these three items from the SDS and recalculating the association of a new SDS index and clinical symptoms of depression. Third, the present study only used different types of coping styles as the variable to examine criterion validity. Consequently, other criteria, such as clinical diagnoses, may be better than the coping style.

## 5. Conclusions

The present study results showed that SDS and SCL-90-R had good psychometric characteristics in the police officer population studied, and they can be used as reliable instruments for assessing police mental health. As for the specification of these two measures, Kitamura et al.’s three-factor SDS model [15] and Arrindell and Ettema’s eight-factor SCL-90-R model [21] are recommended due to their best fit in the CFA results reported here. From the results of the bi-factor model, it is recommended that the scores of cognitive and somatic symptoms of SDS and the scores of anxiety and depression subscale of SCL-90-R should not be used alone because they may cause misdiagnosis of specific mental illness types.

## Figures and Tables

**Table 1:** Descriptive statistics and correlation matrix.

	Mean (SD)	1	2	3	4	5	6	7	8	9	10	11	12	13	14	15	16	17
1. SDS	52.18 (11.25)	1.00																
2. SCL-90-R	174.60 (56.19)	0.66	1.00															
3. SOM	1.89 (0.70)	0.57	0.87	1.00														
4. O-C	2.24 (0.73)	0.60	0.92	0.76	1.00													
5. I-S	1.98 (0.69)	0.56	0.91	0.71	0.83	1.00												
6. DEP	2.09 (0.75)	0.66	0.95	0.78	0.87	0.86	1.00											
7. ANX	1.89 (0.70)	0.62	0.94	0.81	0.85	0.84	0.88	1.00										
8. HOS	1.96 (0.76)	0.57	0.86	0.68	0.75	0.77	0.79	0.78	1.00									
9. PHOB	1.45 (0.53)	0.50	0.81	0.68	0.70	0.75	0.72	0.77	0.67	1.00								
10. PAR	1.86 (0.65)	0.56	0.87	0.67	0.78	0.83	0.81	0.80	0.79	0.67	1.00							
11. PSY	1.78 (0.63)	0.61	0.93	0.76	0.81	0.85	0.86	0.87	0.76	0.75	0.83	1.00						
12. Problem-solving	0.76 (0.21)	−0.42	−0.36	−0.26	−0.34	−0.34	−0.40	−0.34	−0.31	−0.26	−0.31	−0.34	1.00					
13. Self-blame	0.45 (0.29)	0.48	0.52	0.38	0.48	0.51	0.55	0.49	0.44	0.38	0.47	0.49	−0.21	1.00				
14. Help-seeking	0.55 (0.23)	−0.28	−0.26	−0.21	−0.26	−0.22	−0.30	−0.24	−0.20	−0.17	−0.23	−0.24	0.46	−0.14	1.00			
15. Illusion	0.52 (0.24)	0.36	0.42	0.31	0.38	0.41	0.39	0.38	0.40	0.32	0.42	0.40	−0.01 ^a^	0.66	0.05 ^c^	1.00		
16. Avoidance	0.59 (0.24)	0.37	0.40	0.31	0.37	0.39	0.42	0.36	0.33	0.29	0.35	0.37	−0.16	0.64	−0.06 ^d^	0.61	1.00	
17. Rationalization	0.52 (0.21)	0.31	0.34	0.23	0.31	0.34	0.34	0.31	0.29	0.27	0.36	0.33	0.01 ^b^	0.63	0.05 ^e^	0.63	0.64	1.00

Note. The correlation coefficients all reached a significant level of *p* < 0.01, except that the *p*-values of a, b, c, d, and e were 0.68, 0.78, 0.07, 0.06, and 0.10, respectively; SDS = Self-Rating Depression Scale; SCL-90-R = Symptom Checklist 90-Revised; SOM = somatization; O-C = obsessive-compulsive; I-S = interpersonal sensitivity; DEP = depression; ANX = anxiety; HOS = hostility; PHOB = phobic anxiety; PAR = paranoid ideation; PSY means psychoticism.

**Table 2:** Model comparison of SDS and SCL-90-R.

Fit Indices				SDS		Models ^a^	
	One-factor model	Two-factor model [26]	Three-factor model [12]	Three-factor model [15]	Three-factor model [16]	Four-factor model [14]	Bi-factor model with Kitamura et al.’s three-factor model
χ^2^ (df)	3329.71 (170)	910.80 (169)	2812.39 (167)	724.80 (167)	1024.80 (167)	896.95 (160)	618.79 (150)
CFI	0.832	0.961	0.860	0.970	0.954	0.961	0.975
NNFI	0.813	0.956	0.840	0.966	0.948	0.954	0.968
RMSEA	0.127	0.062	0.117	0.054	0.067	0.063	0.052
SRMR	0.118	0.064	0.116	0.066	0.081	0.076	0.065
AIC	3409.71	992.80	2898.39	810.80	1110.80	996.95	1101.70
**Fit Indices**				**SCL-90-R**		**Models ^b^**	
	One-factor model	Nine-factor model	Second-order model (with nine factors) ^c^	Eight-factor model	Second-order model (with eight factors) ^c^	Bi-factor model with nine factors	Bi-factor model with eight factors
χ^2^ (df)	36,903.42 (3320)	26,957.46 (3284)	-- (3311)	24,524.62 (3131)	-- (3151)	25,402.36 (3237)	22,981.36 (3078)
CFI	0.975	0.979	--	0.979	--	0.980	0.981
NNFI	0.974	0.978	--	0.978	--	0.979	0.980
RMSEA	0.093	0.079	--	0.077	--	0.077	0.075
SRMR	0.048	0.047	--	0.046	--	0.043	0.043
AIC	37,235.42	27,361.46	--	24,904.62	--	27,406.41	23,467.36

Note. ^a^ The specifications of each model of the SDS can be seen in the figures in the Supplementary Materials (Figure S1). ^b^ Given that there are many items in SCL-90-R, the specifications of each model are not easy to present graphically. Among them, the factors were correlated in the eight-factor model and nine-factor model. The correlations between the factors of the eight-factor model were described above. The correlations between the factors of the nine factors were between 0.79 and 0.98, and 16 of the 33 correlation coefficients were more than 0.9. As for the bi-factor model, all items were loaded on a general factor of psychological distress, and each item was additionally loaded on a specific factor (e.g., SOM factor of nine-factor model in the SCL-90-R). The specific factors correlated neither with each other nor with the general factor. ^c^ The second-order model with nine-factor and eight-factor models did not converge; CFI = comparative fit index; NNFI = non-normed fit index; RMSEA = root mean square error of approximation; SRMR = standardized root mean square residual; AIC = Akaike information criterion.

**Table 3:** Item properties of SDS.

Items	M (SD)	Loading		
		Without Using Bi-Factor	Specific	General
**Affective Symptoms**				
15. Irritability	1.82 (0.88)	0.66	0.58	0.33
1. Depressed affect	2.14 (0.81)	0.76	0.61	0.43
10. Fatigue	2.42 (0.93)	0.73	0.64	0.35
3.Crying spells	1.48 (0.69)	0.65	0.52	0.37
13. Psychomotor agitation	1.80 (0.84)	0.79	0.64	0.44
9. Tachycardia	1.55 (0.73)	0.62	0.63	0.22
19. Suicidal ideation	1.29 (0.68)	0.53	0.39	0.32
4. Sleep disturbance	2.61 (1.05)	0.54	0.51	0.22
8. Constipation	1.47 (0.78)	0.39	0.42	0.12
**Cognitive Symptoms**				
17. Personal devaluation	2.59 (1.02)	0.65	0.08	0.65
18. Emptiness	2.36 (1.03)	0.82	0.71	0.78
14. Hopelessness	2.52 (1.05)	0.74	0.14	0.71
16. Indecisiveness	2.88 (0.89)	0.58	−0.11	0.62
20. Dissatisfaction	1.75 (0.97)	0.66	0.03	0.63
2. Worse in the morning	2.85 (0.99)	0.29	0.13	0.30
**Somatic Symptoms**				
5. Decreased appetite	2.09 (1.12)	0.56	0.32	0.44
6. Decreased libido	1.81 (1.00)	0.56	0.31	0.43
12. Psychomotor retardation	2.51 (0.95)	0.78	0.39	0.63
11. Confusion	2.28 (1.01)	0.81	0.66	0.60
7. Weight loss	1.55 (0.83)	0.10	−0.02	0.04

**Table 4:** Item properties of SCL-90-R.

Items	M (SD)	Loading		
		Without Using Bi-Factor	Specific	General
**AGO**				
15. Afraid on the street	1.28 (0.71)	0.69	0.35	0.61
25. Afraid to go out alone	1.38 (0.76)	0.71	0.48	0.59
47. Afraid of public transport	1.26 (0.68)	0.71	0.50	0.58
50. Having to avoid things/places/activities	2.07 (1.11)	0.74	0.15	0.74
70. Uneasy in crowds	1.44 (0.77)	0.76	0.40	0.65
75. Nervous when alone	1.35 (0.67)	0.83	0.36	0.73
82. Afraid to faint in public	1.30 (0.69)	0.69	0.17	0.64
**ANX**				
2. Nervousness	2.52 (1.12)	0.70	0.02	0.71
17. Trembling	1.43 (0.77)	0.70	0.14	0.67
23. Suddenly scared	1.56 (0.88)	0.81	0.43	0.77
33. Feeling fearful	1.74 (0.94)	0.84	0.34	0.81
39. Heart pounding/racing	1.87 (0.98)	0.78	0.12	0.75
57. Feeling tense	2.34 (1.08)	0.81	0.01	0.82
72. Spells of terror/panic	1.53 (0.84)	0.83	0.31	0.80
78. Can’t sit still/restless	1.78 (0.94)	0.85	0.03	0.84
80. Something bad is going to happen to you	1.81 (0.96)	0.78	0.05	0.77
86. Frightening thoughts	2.30 (1.09)	0.52	−0.08	0.54
**DEP**				
3. Unpleasant thoughts	2.23 (1.08)	0.69	0.05	0.68
5. Loss of sexual interest	1.98 (1.04)	0.51	−0.01	0.50
14. Low energy/slow	2.88 (1.22)	0.73	0.09	0.71
15. Thoughts of ending life	1.28 (0.71)	0.67	0.03	0.65
19. Poor appetite	1.98 (0.95)	0.59	0.04	0.58
20. Crying easily	1.56 (0.83)	0.61	−0.02	0.62
22. Feeling trapped	1.49 (0.83)	0.71	0.06	0.71
26. Blaming yourself	2.11 (1.03)	0.76	0.09	0.75
29. Feeling lonely	2.30 (1.20)	0.81	0.38	0.77
30. Feeling blue	2.45 (1.18)	0.85	0.64	0.80
31. Worrying too much	2.51 (1.15)	0.82	0.26	0.79
32. No interest in things	2.24 (1.11)	0.75	0.12	0.73
51. Mind going blank	2.08 (1.07)	0.79	0.04	0.78
54. Hopeless about future	2.62 (1.33)	0.75	0.08	0.73
59. Thoughts of death or dying	1.78 (1.05)	0.70	0.06	0.70
79. Feeling worthless	1.92 (1.04)	0.76	−0.03	0.77
**SOM**				
1. Headaches	2.09 (1.01)	0.65	0.30	0.58
4. Faintness	1.58 (0.80)	0.66	0.33	0.57
12. Pains in heart/chest	1.73 (0.97)	0.71	0.38	0.61
27. Pains in lower back	2.46 (1.25)	0.66	0.37	0.56
40. Nausea	1.99 (1.08)	0.72	0.41	0.60
42. Soreness of muscles	2.29 (1.11)	0.74	0.42	0.62
48. Trouble getting breath	1.46 (0.79)	0.79	0.36	0.70
49. Hot/cold spells	1.43 (0.72)	0.77	0.34	0.68
52. Numbness	1.77 (0.98)	0.81	0.50	0.67
53. Lump in throat	1.86 (1.02)	0.73	0.41	0.62
56. Weakness of body	2.21 (1.09)	0.77	0.22	0.74
58. Heavy arms/legs	1.92 (1.04)	0.82	0.31	0.76
**COG**				
9. Trouble remembering	3.01 (1.20)	0.64	0.02	0.62
10. Worried about sloppiness	1.92 (0.90)	0.54	0.01	0.55
28. Feeling blocked	2.44 (1.09)	0.77	0.02	0.76
38. Doing things slowly	2.14 (1.11)	0.65	0.10	0.62
45. Having to double-check	2.36 (1.03)	0.72	0.30	0.68
46. Difficulty deciding	2.45 (1.09)	0.76	0.08	0.74
55. Trouble concentrating	2.35 (1.04)	0.81	0.03	0.79
65. Repeating same actions	1.45 (0.85)	0.57	0.12	0.57
71. Everything is an effort	1.85 (0.93)	0.82	−0.03	0.82
**I-S**				
6. Feeling critical of others	2.15 (0.99)	0.65	0.16	0.62
7. Someone can control your thoughts	1.73 (0.98)	0.68	0.16	0.66
8. Others are to blame	2.18 (0.95)	0.64	0.18	0.61
18. Most people can’t be trusted	1.95 (0.94)	0.77	0.20	0.75
21. Feeling shy opposite sex	1.87 (0.93)	0.57	−0.01	0.57
34. Feeling easily hurt	2.16 (1.08)	0.75	0.10	0.75
35. Others knowing your private thoughts	1.85 (0.82)	0.62	0.22	0.58
36. Others are unsympathetic	1.97 (0.97)	0.82	0.42	0.77
37. People dislike you	1.84 (0.87)	0.83	0.47	0.76
41. Feeling inferior to others	2.20 (1.06)	0.74	0.11	0.73
43. Feeling watched	1.59 (0.84)	0.78	0.31	0.73
61. Uneasy when people are watching you	2.22 (1.02)	0.77	0.10	0.75
68. Having beliefs that others do not share	1.92 (0.89)	0.71	0.04	0.70
69. Self-conscious with others	1.72 (0.87)	0.84	0.13	0.81
73. Uncomfortable eating/drinking in public	1.66 (0.88)	0.56	−0.02	0.56
76. Not getting enough credit	2.10 (1.01)	0.74	0.20	0.71
83. People will take advantage	1.43 (0.74)	0.71	0.21	0.67
88. Never feeling close to another person	1.88 (1.04)	0.69	0.06	0.70
**HOS**				
11. Easily annoyed	2.80 (1.14)	0.77	0.15	0.72
24. Temper outbursts	2.22 (1.12)	0.78	0.16	0.73
63. Urges to harm someone	1.71 (1.02)	0.76	0.37	0.69
67. Urges to break things	1.84 (1.10)	0.83	0.44	0.75
74. Arguing frequently	1.80 (0.85)	0.70	0.29	0.62
81. Shouting/throwing	1.39 (0.76)	0.76	0.42	0.67
**Sleep**				
44. Trouble falling asleep	2.68 (1.29)	0.86	0.43	0.66
64. Awakening in the early morning	2.18 (1.23)	0.61	0.31	0.52
66. Sleep that is restless or disturbed	2.71 (1.33)	0.86	0.72	0.65

## Supplementary Materials

The following are available online at https://www.mdpi.com/1660-4601/17/8/2737/s1, Figure S1: Different factor structures of SDS.
