# Supplementary material for: Assessing Mental Health for China’s Police: Psychometric Features of the Self-Rating Depression Scale and Symptom Checklist 90-Revised
Source: Int J Environ Res Public Health. 2020 Apr 16;17(8):2737. doi: 10.3390/ijerph17082737 (PMC7215956; doi:10.3390/ijerph17082737)
Supplement: Supplementary file 1 [file ijerph-17-02737-s001.pdf]

Supplementary Material

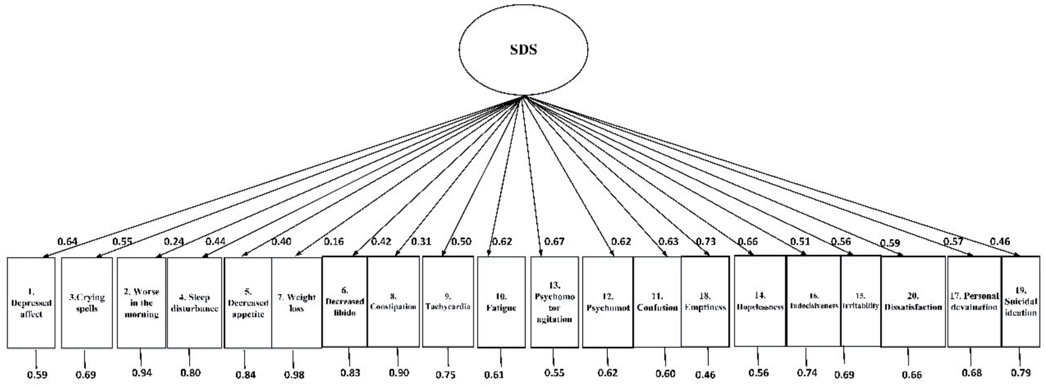

(**a**) SDS: One-factor model

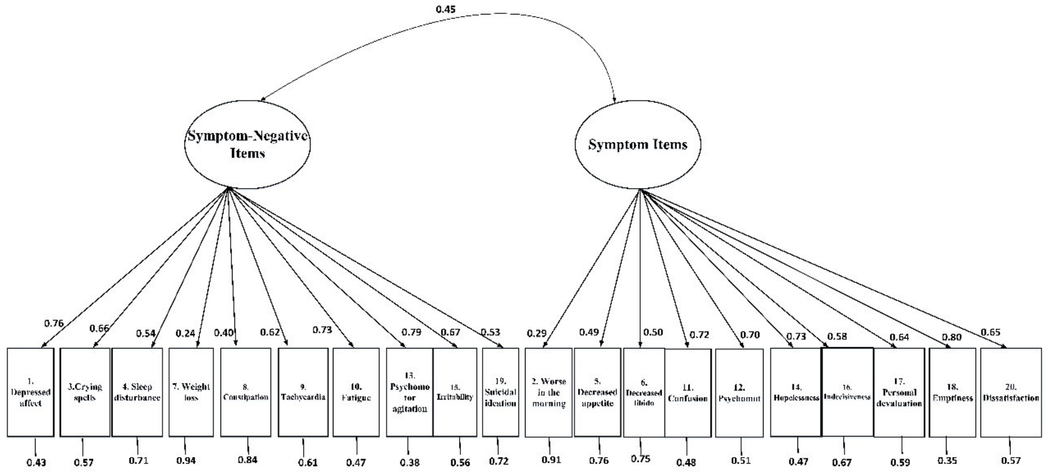

(**b**) SDS: Schotte et al.'s two-factor model

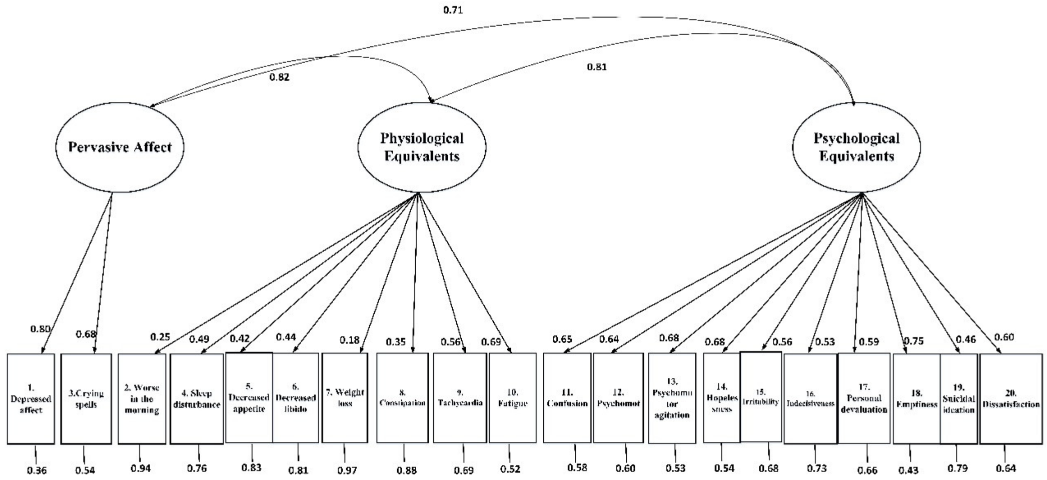

(**c**) SDS: Zung's three-factor model

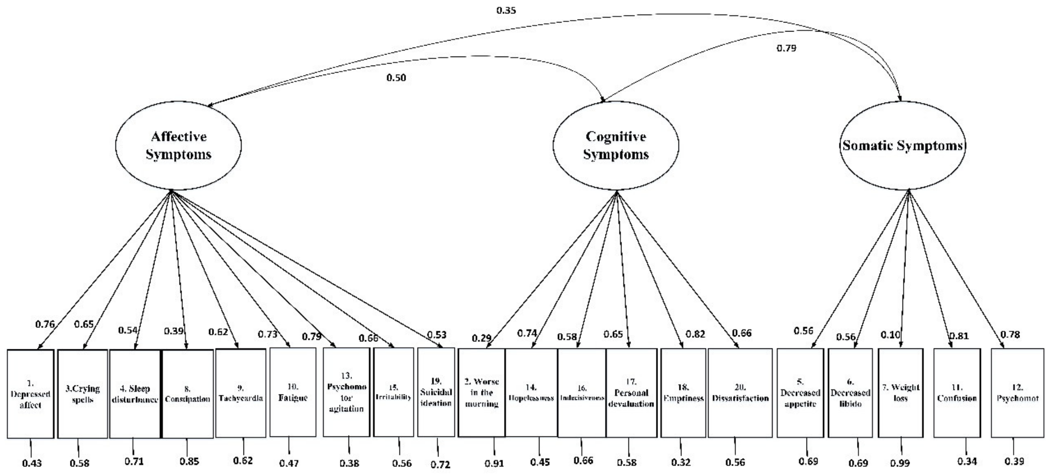

(**d**) SDS: Kitamura et al.'s three-factor model

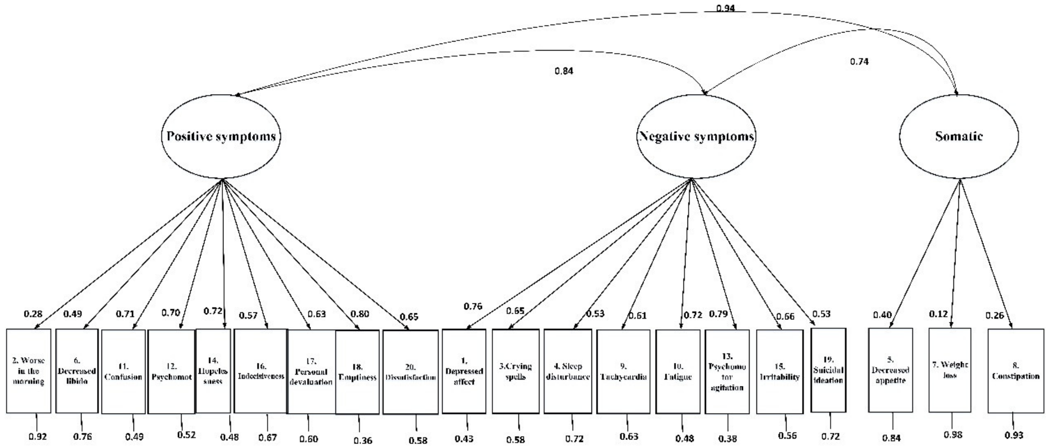

(**e**) SDS: Shafer's three-factor model

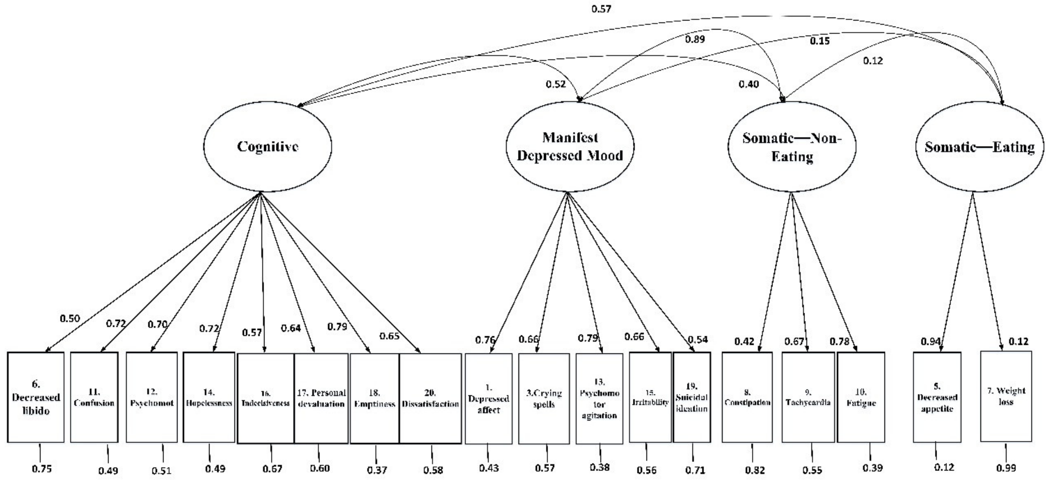

(**f**) SDS: Passik et al.'s four-factor model

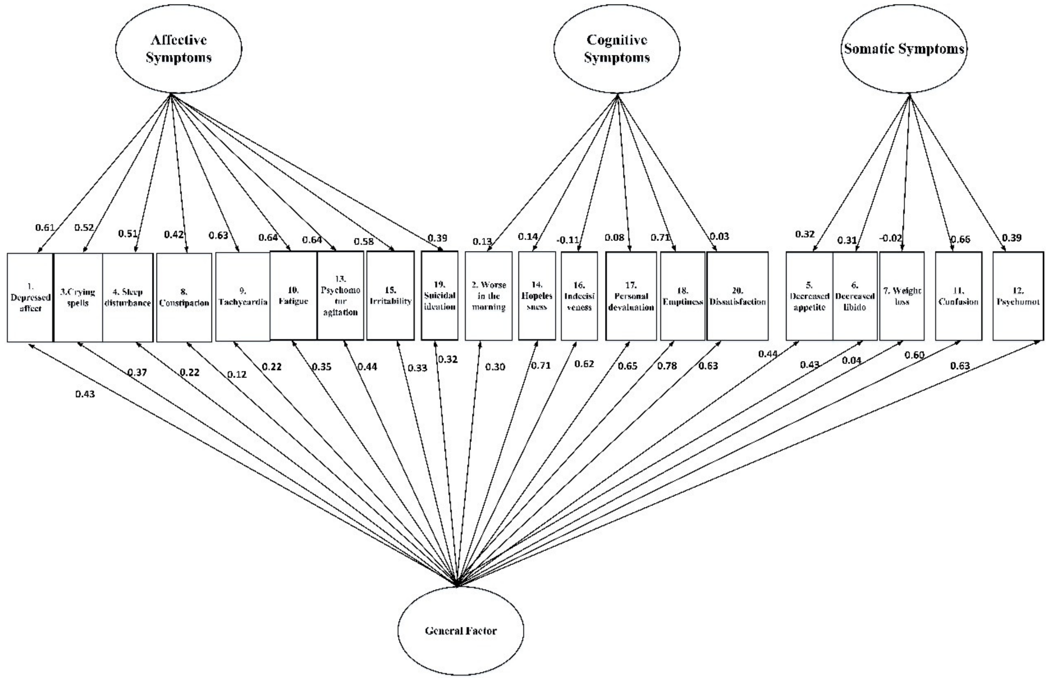

(**g**) SDS: Bifactor model with Kitamura et al.'s three-factor model

Figure S1. Different factor structures of SDS.
